# Pineapple Leaf Phenols Attenuate DSS-Induced Colitis in Mice and Inhibit Inflammatory Damage by Targeting the NF-κB Pathway

**DOI:** 10.3390/molecules26247656

**Published:** 2021-12-17

**Authors:** Yang Chen, Yaoyun Niu, Wenhui Hao, Wanqiu Zhang, Jinghua Lu, Jin Zhou, Lijun Du, Weidong Xie

**Affiliations:** 1 State Key Laboratory of Chemical Oncogenomics, Shenzhen International Graduate School, Tsinghua University, Shenzhen 518055, China; 15555373590@163.com (Y.C.); niuyy1996@163.com (Y.N.); Jasmyn_hao@163.com (W.H.); wanqiuzhangx@126.com (W.Z.); 15813327094@163.com (J.L.); 2 Shenzhen Key Lab of Health Science and Technology, Institute of Biopharmaceutical and Health Engineering, Shenzhen International Graduate School, Tsinghua University, Shenzhen 518055, China; 3 Department of Chemistry, Tsinghua University, Beijing 100084, China; 4 Institute for Ocean Engineering, Shenzhen International Graduate School, Tsinghua University, Shenzhen 518055, China; zhou.jin@sz.tsinghua.edu.cn; 5 Laboratory of Pharmaceutical Science, School of Life Science, Tsinghua University, Beijing 100084, China

**Keywords:** pineapple leaf phenols, caffeic acids, p-coumaric acids, colitis, inflammation

## Abstract

Colitis is not fully curable, although currently, some treatment options are being adopted. In this study, we investigated the effects of pineapple leaf phenols (PLPs), natural phenol products from pineapple leaves, on DSS-induced colitis in mice. The results showed that PLPs dramatically decreased the inflammatory response by inhibiting NF-κB activation and the secretion of pro-inflammatory factors. Moreover, PLPs provided protection against DSS-induced acute colitis by maintaining epithelial integrity. Caffeic and P-coumaric acids had similar effects and could be the active components responsible for PLPs’ effect on colitis. These results indicate that the oral administration of PLPs might be considered as a therapeutic strategy in the treatment of patients with colitis. However, further research on clinical applications and the exact effect of PLPs on colitis is required.

## 1. Introduction

Crohn’s disease (CD) and ulcerative colitis (UC), the two major clinically defined forms of inflammatory bowel diseases (IBDs), are chronic remittent or progressive disorders of the gastrointestinal tract, and it is estimated that there is a prevalence of 250 cases per 100,000 individuals in Western countries [1,2]. Both CD and UC are characterized by intestinal inflammation and epithelial injury and are mediated by shared and distinct inflammatory pathways [3]. The etiology of IBDs is still not fully understood, but it is widely acknowledged that they result from the interaction between genetic predisposition, environmental factors, gut microbiota, and immune response [4].

The dextran sulfate sodium (DSS)-induced colitis model is the most widely used model of colitis, as beyond its shared characteristics with human IBD etiology, pathogenesis, and therapeutic response, it is relatively simple, quick to set up, and cost effective. DSS is a sulfated polysaccharide; the exact mechanisms through which DSS induces intestinal inflammation are unclear but may be the result of direct damage of the monolayer of epithelial cells in the colon, leading to the crossing of intestinal contents (e.g., commensal bacteria and their products) into underlying tissue and, therefore, induction of inflammation. Clinical manifestations of colitis usually include watery diarrhea, occult blood in stools, and weight loss.

Nevertheless, despite the relevance and the impact of the disease on the health and social life of patients, the etiology of UC remains unclear, and the current treatment options, which include corticosteroids, aminosalicylates, immunomodulators, and monoclonal antibodies, often lack clinical effectiveness and have manifold detrimental side effects. Thus, the development of new treatment approaches and management strategies to treat patients according to the severity and extent of UC requires urgent exploration [5].

Taking the above into account, recent studies have shown that medicinal plant-derived extracts, herbs, and dietary components (such as flavonoids) have anti-colitis activity, as they can control the levels of inflammatory mediators associated with the severity of active UC [6,7,8]. PLPs are natural phenol extracts derived from pineapple leaves. These natural extracts have anti-diabetic, anti-oxidative, and lipid-regulating properties [9,10,11,12]. PLPs contains two monomers, caffeic acid and P-coumaric acid. Caffeic acid has been found to provide a certain level of anti-inflammatory protection in DSS-induced colitis in mice and to increase the Akkermansia population in the gut microbiota at a 1 mM concentration [13,14]. It has been proven that P-coumaric acid can achieve anti-inflammatory effects by inhibiting the activation of the MAPK and NF-κB signaling pathways, and the monomer also has anti-oxidative properties [15,16].

Thus, the purpose of our study was to investigate the protective effects of PLPs, Caffeic acid and P-coumaric acid in an acute experimental model of intestinal inflammation chemically induced by dextran sulfate sodium (DSS) in mice. Furthermore, to substantiate the possible effects on barrier function and anti-inflammation, we employed in vitro assays using heterogeneous human epithelial colorectal adenocarcinoma (Caco-2) and RAW264.7 cells.

## 2. Materials and methods

### 2.1. Preparation of Extracts and Active Components

PLPs were prepared according to a previous method [9]. More than 50% of the components of PLPs are phenols, among which Caffeic acid (CAS:331-39-5, Sigma-Aldrich, Chicago, IL, USA) and P-coumaric acid (CAS:501-98-4, Sigma-Aldrich, Chicago, IL, USA) are the two main active components. 

### 2.2. DSS-Induced Colitis Mouse Models

C57BL/6J mice (body weight, 18 ± 2 g; 4-weeks-old; male) were purchased from Guangzhou Medical Animal Centre (Guangzhou, China) and housed under controlled conditions (constant temperature: 22 ± 2 °C; constant humidity: 60 ± 5%; 12 h dark/light cycle). The study was performed in strict accordance with the National Institutes of Health Guide for the Care and Use of Laboratory Animals, and the protocol was approved by the Bioethics Committee of Shenzhen International Graduate School, Tsinghua University, China (Ethics issue (2020) No. 38). The animals were challenged with 3% DSS (g/mL, dextran sulphate sodium, molecular weight: 500,000 Da, Wuhan Yeasen Biotechnology Co., Ltd., Wuhan, China) solution in drinking water for 7 consecutive days, which was replenished daily.

### 2.3. Disease Activity Index (DAI) Assay

Animals were divided into the following treatment groups: (i) normal control group, which received only drinking water; (ii) DSS group treated with vehicle (deionized water, 10 mL/kg, intragastric); (iii) DSS group treated with PLPs (200 mg/kg, intragastric); (iv) DSS group treated with Caffeic acid (50 mg/kg, intragastric); and (v) DSS group treated with P-coumaric acid (50 mg/kg, intragastric). Individual mice were monitored daily to determine the disease activity index (DAI), according to changes in weight loss, stool consistency, and occult blood.

The scores were described as follows: weight loss was graded as 0 if body weight increased or remained within 1% of baseline; 1 for a 1–5% loss; 2 for a 5–10% loss; 3 for a 10–15% loss; or 4 for weight loss > 15%. The stool consistency was graded as 0 for no diarrhea; 2 for loose stool that did not stick to the anus; and 4 for liquid stool that did stick to the anus. The presence of fecal blood received a value of 0 for none, 2 for moderate, and 4 for gross bleeding.

After the experiments, the animals were sacrificed by anesthetizing them with urethane (solution: 10 g per 100 mL saline, 10 mL solution /kg) and subjecting them to cervical dislocation. The colons were collected and washed with saline (0.9%) and the lengths were measured. A portion of colon tissues were used for regular histological analysis. Another portion of the tissues were instantly frozen by using liquid nitrogen and stored at −80 °C for further biochemical analysis.

### 2.4. Histological Analysis

For analysis of microscopic damage, the distal portion of each colon was excised and immediately fixed in 10% formaldehyde, embedded in paraffin wax, and then sectioned at a 7 μm thickness before being deparaffinized. Slides were stained using hematoxylin and eosin stain (H&E) to analyze the histopathological changes.

### 2.5. MPO Activity Assay

The function of myeloperoxidase, which is unique to neutrophils, is to convert chlorine oxide and hydrogen oxide into hypochloric acid. In the inflammatory state, it is released into the extracellular fluid and enters the circulation. Myelinated peroxygenase exists in neutrophils, and each cell contains a certain amount of the enzyme. Therefore, the number of neutrophils can be quantitatively determined. Myeloperoxidase (MPO) activity assay kit (Nanjing Jiancheng Bioengineering Institute, Nanjing, China) was used to measure MPO activity in the homogenates.

### 2.6. Cell Culture

RAW264.7 and human epithelial colorectal adenocarcinoma (Caco-2) cells were provided by the Cell Resource Center of the Shanghai Institute for Biological Science, Chinese Academy of Sciences, China. RAW264.7 cells were cultured in Dulbecco’s modified Eagle’s medium (high glucose, Gibco^®^, Thermo Fisher Scientific, Waltham, MA, USA), supplemented with 10% fetal bovine serum (Premium, Pan Biotech, Adenbach, Germany and 1% pen–strep antibiotics (Gibco^TM^, Thermo Fisher Scientific, Waltham, MA, USA) and then incubated in a humidified atmosphere of 5% CO_2_ at 37 ℃. Caco-2 cells were cultured in Dulbecco’s modified Eagle’s medium (high glucose, Gibco^®^, Thermo Fisher Scientific, Waltham, MA, USA), supplemented with 20% fetal bovine serum (Premium, Pan Biotech, Adenbach, Germany) and 1% pen–strep antibiotics (Gibco^TM^, Thermo Fisher Scientific, Waltham, MA, USA) and then incubated in a humidified atmosphere of 5% CO_2_ at 37 ℃.

### 2.7. In Vitro Scratch Assay

To evaluate intestinal mucosal healing, we used a classic in vitro wound healing assay, which consists of creating a “scratch” in a cell monolayer. Caco-2 cells were grown to confluence (3 days) on six-well plates (TCP-010-006, Guangzhou Jet Bio Filtration Co., Ltd., Guangzhou, China) at a density of 2.5 × 10^5^ cells per well. A linear scratch was made in each well using a 200 μL sterile pipette plastic tip, perpendicular to a black line drawn on the underside of the plate for reference, creating a cell-free area. Posteriorly, to remove detached cells and debris, the wounded monolayer was washed with PBS and incubated in 2 mL of FBS-free culture medium containing PLPs (20 μg/mL dissolved in DMSO), caffeic acid (10 μg/mL dissolved in DMSO), and P-coumaric acid (10μg/mL) at 37 °C. PLPs, caffeic acid, and p-coumaric acid were dissolved in DMSO and a vehicle control was added at a volume identical to that of DMSO. Images of each scratch were captured at 0, 24, and 48 h with a digital camera on an inverted microscope (Leica Microsystems, Weztlar, Germany) at 50× magnification. Wound closure analysis was conducted from edge to edge using the ImageJ software. For all treatments, the wound at 0 h was assigned as 100%, and the percentages of wound healing at 24 and 48 h were compared to each cell treatment at 0 h.

### 2.8. Inflammation Induction

RAW264.7 cells were seeded into six-well plates at a density of 2.5 × 10^5^ cells per well. Inflammation was induced by adding 1 ug/mL lipopolysaccharide (LPS, Lot. No. L4391, Sigma-Aldrich, Chicago, IL, USA) after 12 h of attachment. PLPs (20 μg/mL), caffeic acid (10 μg/mL), and P-coumaric acid (10 μg/mL) were added, and the vehicle control was added at a volume identical to that of DMSO. Cell samples were collected for further analysis after 12 h of treatment.

### 2.9. Western Blotting Analysis

Total proteins were extracted from mouse colon tissues or RAW264.7 cells with lysis buffer. The protein samples were separated using 12.5% SDS-PAGE and then transferred to PVDF membranes (Pall, New York, NY, USA). Subsequently, the membranes were blocked with blocking buffer (5% skimmed milk power (Anchor, Auckland, New Zealand), which was dissolved in TBS containing 0.5% Tween-20 (TBST; Sangon Biotech Co., Ltd., Shanghai, China)) for 2 h, and then incubated with primary antibodies dissolved in 3% BSA overnight at 4 ℃. After the membrane was washed with TBST buffer (TBS added with 0.5% Tween-20) three times, secondary antibodies (dissolved in blocking buffer) were added and incubated for 2 h. After washing again, the protein bands were visualized using enhanced chemiluminescence (Pierce^TM^ ECL Western Blotting Substrate; Thermo Fisher Scientific, Inc., Waltham, MA, USA). The following primary antibodies were used: anti-GAPDH (1:10,000; A01020; Abbkine Scientific Co., Ltd., Wuhan, China); anti-p65 (1:2000, #3033, Cell Signaling Technology, Boston, MA, USA); anti-phospho-p65 (1:2000, #3039, Cell Signaling Technology, Boston, MA, USA); and IL-1β (1:2000, #12242, Cell Signaling Technology, Boston, MA, USA).

### 2.10. Reverse Transcription and Quantitative Real-Time Polymerase Chain Reaction (RT-qPCR) Assay

Total RNA was extracted from mouse colon tissues, RAW264.7, or Caco-2 samples by using AG RNAex Pro Reagent (AOAKR, Code#:AG21102, Changsha, China), according to the protocol of the kit. Approximately 500 ng total RNA was reverse transcribed into cDNA using the Evo M-MLV RT Premix for the qPCR kit (Accurate Biotechnology, Hunan, China). The RT procedure was performed with a thermocycler program comprising the following steps: 37 °C for 15 min, 85 °C for 5 s, and a hold step at 4 °C. qPCR was performed using SYBR Green Premix Pro Taq HS qPCR kit (Accurate Biotechnology, Hunan, China). The primers were synthesized by Genewiz, Inc., Suzhou, China (Table 1). The qPCR procedure involved pre-denaturation of cDNA samples at 95 °C for 30 s and the amplification of the denatured cDNA samples with 40 cycles at 95 °C for 5 s and at 60 °C for 30 s. Data were normalized using the β-actin gene, and fold changes were calculated using the 2-ΔΔCt normalization method.

### 2.11. Statistical Analysis

Statistical analysis was conducted using GraphPad Prism 8 (GraphPad software Inc., Waltham, MA, USA). Data are expressed as mean ± standard deviation (S.D.). Differences with statistical significance between groups were calculated by ANOVA followed by Tukey’s post hoc test. *p* < 0.05 was considered statistically significant.

## 3. Results

### 3.1. Treatment with PLPs and Their Monomers Caffeic Acid and P-Coumaric Acid Ameliorates the Symptoms of DSS-Induced Experimental Colitis in Mice

**Figure 1:**
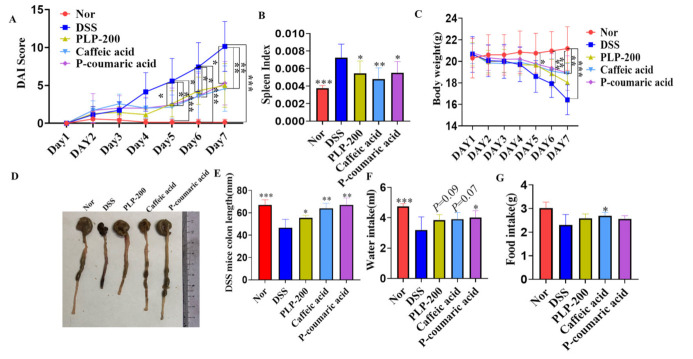
PLPs, Caffeic acid, and P-coumaric acid treatment ameliorated the symptoms of DSS-induced experimental colitis in mice (**A**–**G**). Disease activity index (DAI) (**A**). Spleen index (**B**). Loss of basal body weight (**C**). Colon length measured for each group (**D**,**E**). Water intake and food intake (**F**,**G**). Nor: normal control mice; DSS: colitis model control mice fed dextran sulphate sodium salt; PLP-200: pineapple leaf phenols-treated (200 mg/kg) DSS mice; Caffeic acid: Caffeic acid treated (50 mg/kg) DSS mice; P-coumaric acid: P-coumaric acid-treated (50 mg/kg) DSS mice. Data are expressed as the mean ± SD (n = 7); * *p* < 0.05, ** *p* < 0.01, *** *p* < 0.001 compared to the mice treated with DSS alone.

In the DSS animal experiment, 3% (*w*/*v*) DSS was dissolved in drinking water and provided to C57 mice for 7 days. After treatment, PLPs, caffeic acid, and P-coumaric acid clearly ameliorated DAI (Figure 1A) and lowered the spleen index (Figure 1B) but did not have a significant effect on weight loss (Figure 1C) in the DSS-induced mouse model. Meanwhile, in the PLPs, Caffeic acid, and P-coumaric acid administration groups, the colon length significantly recovered from DSS damage (Figure 1D,E). Indeed, the water intake and food intake of the PLPs, Caffeic acid, and P-coumaric acid administration groups were higher than those in the model group, which suggests that PLPs, Caffeic acid, and P-coumaric acid can slow down intestinal damage caused by DSS to some extent (Figure 1F,G).

### 3.2. PLPs, Caffeic Acid, and P-Coumaric Acid Administration Reduces Histopathological Damage and Inflammatory Response in DSS-Induced Colitis

**Figure 2:**
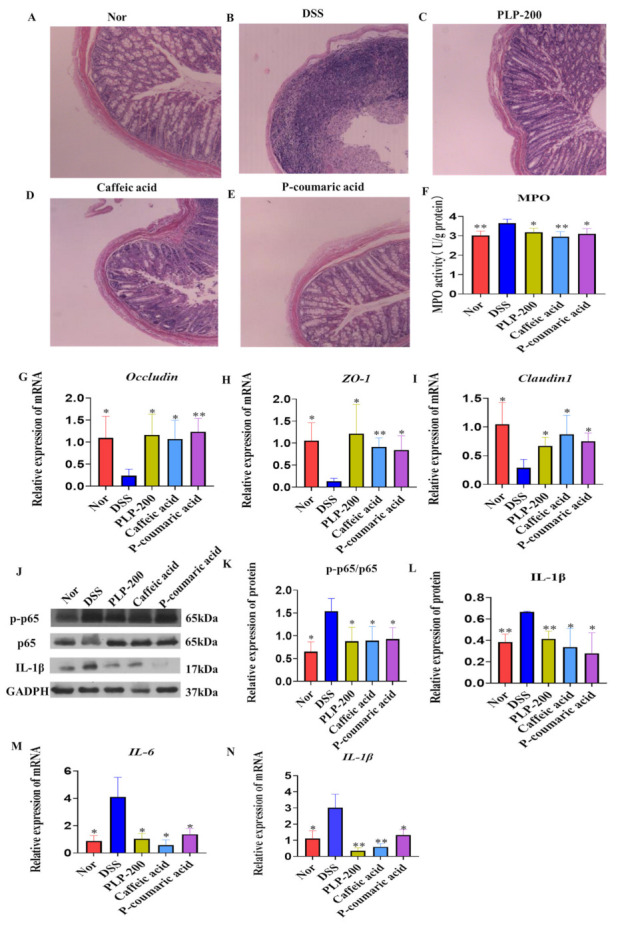
PLP, Caffeic acid, and P-coumaric acid slowed down the loss of tight junction proteins in colonic epithelial tissue and the infiltration of immune cells, and they inhibited the secretion of pro-inflammatory factors and the activation of NF-κB cell signaling in the colon tissue of DSS-induced colitis mice (**A**–**N**). Representative histopathological images at 100× magnification (**A**–**E**). Colonic MPO activity (**F**). ZO-1, occludin, and claudin 1 mRNA in colon tissue were determined by real-time PCR (**G**–**I**). p-p65, p65, and IL-1b were detected by Western blot (**J**–**L**). IL-6 and IL-1β mRNA in colon tissue were determined by real-time PCR (**M**,**N**). Nor: normal control mice; DSS: colitis model control mice fed dextran sulphate sodium salt; PLP-200: pineapple leaf phenols-treated (200 mg/kg) DSS mice; Caffeic acid: Caffeic acid-treated (50 mg/kg) DSS mice; P-coumaric acid: P-coumaric acid-treated (50 mg/kg) DSS mice. Data are expressed as the mean ± SD (n = 7); * *p* < 0.05, ** *p* < 0.01 compared to the mice treated with DSS alone.

To further confirm the role of PLPs, caffeic acid, and P-coumaric acid on inflammation and tissue injury, colonic sections of different groups were histologically stained and examined. Colon tissue of different groups were harvested and homogenated to detect MPO activity, mRNA levels of occludin, ZO-1, and claudin 1. In the DSS group, there was severe and diffuse destruction of the epithelial layer, and the epithelium and lamina propria of the colon showed extensive inflammatory cell infiltration. Compared to the DSS group, PLPs, Caffeic acid, and P-coumaric acid treatment led to much less infiltration of inflammatory cells and tissue damage (Figure 2A–E). It was found that PLPs, caffeic acid, and P-coumaric acid clearly reduced MPO activity (Figure 2F) as well as reducing the mRNA levels of occludin, ZO-1, and claudin 1 in the colon tissue compared to the DSS group (Figure 2G–I).

Furthermore, we detected mature IL-1β, p-p65, and p65 activity (Figure 2J–L) as well as mRNA levels of IL-6 and IL-1β (Figure 2M,N). It was found that PLPs, Caffeic acid, and P-coumaric acid clearly reduced the expression of protein levels of IL-1β, the phosphorylation of p65, and mRNA levels of IL-1β and IL-6 in colon tissues compared to the DSS group. The decrease in mature IL-1β was clear in the PLPs, Caffeic acid, and P-coumaric acid administration groups, which may be attributed to the inhibitory effect of PLPs and their monomers on NF-κB activation. These results indicate that the administration of PLPs and their monomers Caffeic acid and P-coumaric acid can reduce intestinal mucosal damage and ameliorate DSS-induced severe colitis.

### 3.3. PLPs, Caffeic Acid, and P-Coumaric Acid Inhibit the Secretion of Pro-Inflammatory Factors and the Activation of NF-κB Cell Signaling in LPS-Stimulated RAW264.7 Cells

**Figure 3:**
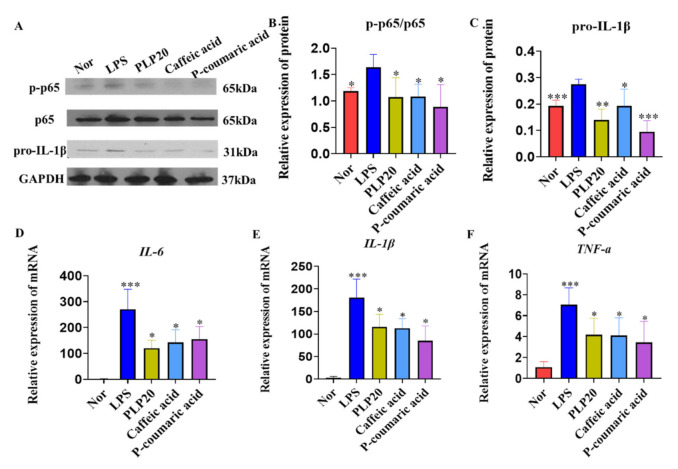
PLPs, Caffeic acid, and P-coumaric acid inhibited the secretion of pro-inflammatory factors and the activation of NF-κB cell signaling in LPS-stimulated RAW264.7 cells. RAW264.7 cells were stimulated with LPS (1 µg/mL) and treated with PLPs (20 μg/mL), Caffeic acid (10 μg/mL), and P-coumaric acid (10 μg/mL) for 12 h (**A**–**F**). p-p65, p65, and IL-1β were detected by Western blot (**A**–**C**). IL-6, IL-1β, and TNF-α mRNA in colon tissue were determined by real-time PCR (**D**–**F**). Nor: normal control cells; LPS: lipopolysaccharide-induced control cells; PLP20: pineapple leaf phenols-treated (20 μg/mL) LPS-induced cells; Caffeic acid: Caffeic acid-treated (10 μg/mL) LPS-induced cells; P-coumaric acid: P-coumaric acid-treated (10 μg/mL) LPS-induced cells. Data are expressed as the mean ± SD, and the results are representative of at least three independent experiments. * *p* < 0.05, ** *p* < 0.01, *** *p* < 0.001 compared to the cells treated with DSS alone.

To investigate the anti-inflammatory effect of PLPs, Caffeic acid, and P-coumaric acid on immune cells, we used LPS-stimulated RAW264.7 cells for 12 h, and then we detected pro-IL-1β, p-p65, and p65 activity (Figure 3A–C) and mRNA levels of IL-1β, IL-6 and TNF-α (Figure 3D–F). The results showed that PLPs, Caffeic acid, and P-coumaric intensely inhibited the secretion of pro-IL-1β and phosphorylation of p65 (Figure 3A–C). Consistently, PLP, caffeic acid, and P-coumaric acid clearly reduced the mRNA levels of IL-1β, IL-6, and TNF-α compared to the LPS group (Figure 3D–F). These data suggest that PLPs, caffeic acid, and P-coumaric acid had a direct anti-inflammatory effect.

### 3.4. PLPs, Caffeic Acid, and P-Coumaric Acid Accelerate Wound Closure and Increase the Gene Expression of Occludin, ZO-1, and Claudin 1 in Caco-2 Cells

**Figure 4:**
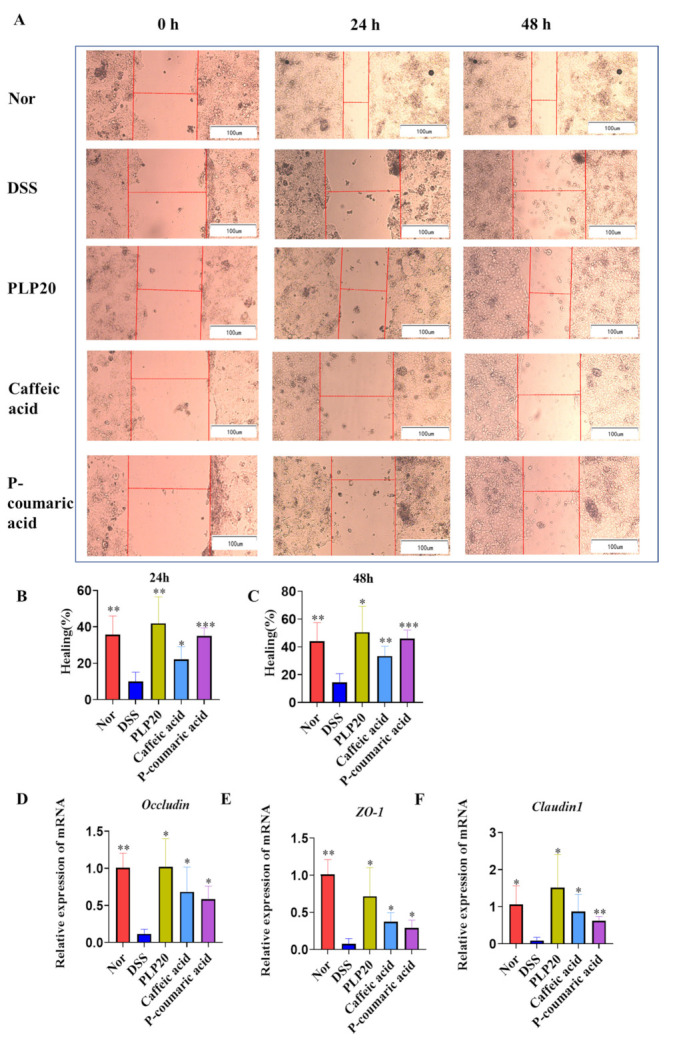
PLP, Caffeic acid, and P-coumaric acid accelerate the wound closure and increase the gene expression of occluding, ZO-1, and clauding1 in Caco-2 cells. Caco-2 cells were stimulated with DSS (3%) and treated with PLPs (20 μg/mL), Caffeic acid (10 μg/mL), and P-coumaric acid (10 μg/mL) (**A**–**F**). Representative images of scratched areas at 0, 24, and 48 h; bar = 100 μm (n = 4) (**A**). Percentage of wound healing at 24 and 48 h after scratch (n = 4) (**B**,**C**). Occludin, ZO-1, and claudin 1 mRNAs were determined by real-time PCR (**D**–**F**). Nor: normal control cells; DSS: dextran sulphate sodium salt-induced control cells; PLP20: pineapple leaf phenols-treated (20 μg/mL) DSS-induced cells; Caffeic acid: caffeic acid-treated (10 μg/mL) DSS-induced cells; P-coumaric acid: P-coumaric acid-treated (10 μg/mL) DSS-induced cells. Data are expressed as the mean ± SD, and the results ae representative of at least three independent experiments. * *p* < 0.05, ** *p* < 0.01, *** *p* < 0.001 compared to the cells treated with DSS alone.

We also determined whether PLPs, Caffeic acid, and P-coumaric acid were capable of accelerating wound healing in Caco-2 cells in vitro. PLPs, Caffeic acid, and P-coumaric acid greatly accelerated the wound closure of DSS-induced and scratched Caco-2 cell monolayers after 24 and 48 h (Figure 4A–C). The results show that PLPs, Caffeic acid, and P-coumaric acid promoted lesion closure by 38.45%, 25.75%, and 35.02% at 24 h and 47.9%, 36.13%, and 45.98% at 48 h, respectively, when compared to the DSS group. Meanwhile, PLPs, Caffeic acid, and P-coumaric acid can effectively increase the mRNA levels of occludin, ZO-1, and claudin 1 (Figure 4D–F).

All of these data indicate that PLPs treatment ameliorates the symptoms of DSS-induced experimental colitis in mice. PLPs, Caffeic acid, and P-coumaric acid promoted intestinal mucosal repair and protected intestinal barrier function. Meanwhile, PLPs, Caffeic acid, and P-coumaric acid provided protection against DSS-induced colitis in mice by inhibiting the secretion of pro-inflammatory factors and the activation of NF-κB to a certain extent.

## 4. Discussion

The DSS model is characterized by a general inflammatory process directly associated with body weight loss, bloody diarrhea, and histopathologic changes that mimic some clinical aspects of UC in humans [17]. Indeed, we observed that DSS induced body weight loss and caused a significant increase in the DAI score, which was accompanied by colonic shortness and bloody stool. Interestingly, PLPs, Caffeic acid, and P-coumaric acid treatment markedly improved these features in mice with DSS-induced colitis, showing that PLPs, Caffeic acid, and P-coumaric acid might constitute a potential alternative to treatment with the aim of preventing or delaying the progression of inflammatory conditions in the gut. Currently, conventional therapy for UC, such as glucocorticoids, 5-ASA, immunomodulators, and biological agents, is used in the active phase and to maintain remission. However, the adverse effects remain a major concern for prolonged usage, which may also include poor responders, peptic ulceration, and wound healing impairment [18,19,20]. In this sense, treatment with PLPs, Caffeic acid, and P-coumaric acid can improve the general parameters of the disease in the DSS model, suggesting that PLPs, Caffeic acid, and P-coumaric acid could be an appropriate option for the treatment of UC, since these extracts and compounds come from natural plants. Furthermore, PLPs, which are natural extracts, were found to improve colitis in mice for the first time.

One of the most typical events underlying the pathogenesis of IBD is the influx of inflammatory cells to the intestinal mucosa. We found that PLPs, Caffeic acid, and P-coumaric acid significantly reduced MPO activity in the colitis model; thus, the anti-inflammatory effects presented by PLPs, Caffeic acid, and P-coumaric acid could be evidenced by the significant reduction in intraepithelial immune cell infiltration. Meanwhile, microscopic examination of colon sections revealed marked intestinal epithelial destruction caused by DSS, with histopathological changes in the mucosa, submucosa, muscular layer and colonic wall, but this was reversed in PLP-treated, Caffeic acid-treated and P-coumaric acid-treated mice.

It is widely believed that the destruction of the intestinal epithelial TJ barrier leads to increased intestinal permeability and plays a key role in the progress of IBD. TJ proteins include ZO-1, occludin, and claudin 1, which play a critical role in maintaining the intestinal epithelial barrier function under oxidative stress or inflammatory conditions, because they mechanically seal the transparency between IECs [21,22,23]. Notably, experimental data of the colon biopsies of IBD patients highlighted an impairment of the intestinal barrier accompanied by decreased expression of TJ proteins [24,25]. In the present study, we found that PLPs, Caffeic acid, and P-coumaric acid can effectively inhibit the decreased expressions of TJ mRNAs, which means PLPs, Caffeic acid, and P-coumaric acid can protect the intestinal barrier by regulating TJ mRNA levels. However, TJ proteins should be investigated in a future study. To confirm the protective effects of PLPs, Caffeic acid, and P-coumaric acid, we initiated complementary in vitro experiments using Caco-2 cells. The intestinal epithelium is constantly exposed to an enormous diversity of stimuli, and the lesion of the intestinal epithelial cells is constant and almost unavoidable. In patients with IBD, the intestinal lesion is typical, and during this process, the mucosal cells migrate to cover the injured area, regardless of proliferation, in an attempt to maintain the integrity of the intestinal barrier [7]. Using a classic in vitro cicatrization model conducted with Caco-2 cells, we demonstrated that PLPs, Caffeic acid, and P-coumaric acid treatment facilitated the cicatrization and/or closure process of cell monolayers, and this may be associated with the proliferation or migration of epithelial intestinal cells, giving support to the in vivo data and reinforcing the potential application of PLPs, Caffeic acid, and P-coumaric acid in the tissue healing process.

Numerous pieces of evidence have revealed that the NF-κB pathway plays a critical role in the pathogenesis and progression of IBD [26,27]. LPS stimulation triggers IκBα phosphorylation and ubiquitination, which consequently results in IκBα degradation and the translocation of p65 (a NF-κB protein) from the cytoplasm to the nucleus. Simultaneously, IKK complex activation induced by inflammatory stimulation results in the release of the active protein kinase A catalytic subunit and the phosphorylation of p65 [28]. Phosphorylated p65 triggers a conformational change and has a lower affinity for IκBα, which results in increased nuclear translocation and accumulation of p65. Phosphorylated p65 also promotes its interaction with CBP/p300 and thereby increases p65 transcriptional activity. In the nucleus, p65 upregulates the transcription of pro-inflammatory mediators and cytokines, for instance, IL-1β, IL-6, and TNF-α. In the present study, we observed that PLPs, caffeic acid, and P-coumaric acid can effectively inhibit the secretion of pro-inflammatory factors and the activation of NF-κB cell signaling in colitis tissues. Meanwhile, stimulating RAW264.7 cells with LPS, we found that PLPs, Caffeic acid, and P-coumaric acid can also effectively inhibit pro-inflammatory factors at the protein and mRNA levels and inhibit the activation of NF-κB by inhibiting p65 phosphorylation. Thus, PLPs, Caffeic acid, and P-coumaric have a certain anti-inflammatory effect and provide protection against DSS-induced colitis by reducing its inflammatory effects.

In conclusion, the current research showed that PLPs dramatically decreased the inflammatory response by inhibiting NF-κB activation and the secretion of pro-inflammatory factors. Meanwhile, PLPs may provide protection against DSS-induced acute colitis by maintaining epithelial integrity. Caffeic acid and P-coumaric acid may be the active components of PLPs. Although PLPs, Caffeic, acid and P-coumaric acid showed comparable activities, low-cost PLPs might have more advantages than Caffeic acid or P-coumaric acid alone. Overall, these results indicated that the use of PLPs could be a therapeutic strategy for inflammation-associated disorder management in IBD patients. However, further research on the clinical applications and the exact effect of PLPs on colitis is required.

## Figures and Tables

**Table 1:** Primers for mRNA qPCR.

Gene	Forward	Reverse	NCBI	Size
β-Actin (Mouse)	GTGACGTTGACATCCGTAAAGA	GCCGGACTCATCGTACTCC	NM_007393	245
IL-1β (Mouse)	GCAACTGTTCCTGAACTCAACT	ATCTTTTGGGGTCCGTCAACT	NM_008361	89
IL-6 (Mouse)	CTGCAAGAGACTTCCATCCAG	GAGTGGTATAGACAGGTCTGTTGG	NM_031168	131
TNF-α (Mouse)	GGGCTTCCAGAACTCCA	GCTACAGGCTTGTCACTCG	NM_013693.2	213
ZO-1 (Mouse)	GCCGCTAAGAGCACAGCAA	TCCCCACTCTGAAAATGAGGA	NM_001163574	134
Claudin 1 (Mouse)	GGGGACAACATCGTGACCG	AGGAGTCGAAGACTTTGCACT	NM_016674	100
Occluding (Mouse)	TTGAAAGTCCACCTCCTTACAGA	CCGGATAAAAAGAGTACGCTGG	NM_008756	129
β-Actin (Human)	CATGTACGTTGCTATCCAGGC	CTCCTTAATGTCACGCACGAT	NM_001101	250
ZO-1 (Human)	CAACATACAGTGACGCTTCACA	CACTATTGACGTTTCCCCACTC	NM_003257	105
Claudin 1 (Human)	CCTCCTGGGAGTGATAGCAAT	GGCAACTAAAATAGCCAGACCT	NM_021101	145
Occluding (Human)	ACAAGCGGTTTTATCCAGAGTC	GTCATCCACAGGCGAAGTTAAT	NM_001205254	89

## Data Availability

The data presented in this study are available on the request from the corresponding author.

## Sample Availability

Samples of the pineapple leaf phenols (PLP) are available from authors.
